# CXCR2 expression on granulocyte and macrophage progenitors under tumor conditions contributes to mo-MDSC generation via SAP18/ERK/STAT3

**DOI:** 10.1038/s41419-019-1837-1

**Published:** 2019-08-08

**Authors:** Xiaoqing Han, Huifang Shi, Yingying Sun, Chao Shang, Tao Luan, Dake Wang, Xueqing Ba, Xianlu Zeng

**Affiliations:** 1 The Key Laboratory of Molecular Epigenetics of Ministry of Education, Institute of Genetics and Cytology, School of Life Science, Northeast Normal University, Changchun, Jilin China; 2 People’s Hospital of Rizhao, Rizhao, China

**Keywords:** Cancer microenvironment, Immune evasion

## Abstract

Myeloid-derived suppressor cells (MDSCs) comprise a critical component of the tumor environment and CXCR2 reportedly plays a key role in the pathophysiology of various inflammatory diseases. Here, CXCR2 expression on granulocyte and macrophage progenitor cells (GMPs) was found to participate in myeloid cell differentiation within the tumor environment. In CXCR2-deficient tumor-bearing mice, GMPs exhibited fewer macrophage and dendritic cell progenitor cells than wild-type tumor-bearing mice, thereby decreasing monocytic MDSCs (mo-MDSCs) expansion. CXCR2 deficiency increased SAP18 expression in tumor-bearing mice, which reduced STAT3 phosphorylation through restraining ERK1/2 activation. Our findings reveal a critical role for CXCR2 in regulating hematopoietic progenitor cell differentiation under tumor conditions, and SAP18 is a key negative regulator in this process. Thus, inhibiting CXCR2 expression may alter the tumor microenvironment and attenuate tumor progression.

## Introduction

Myeloid cells constitute the most abundant hematopoietic cells and are important for adaptive immunity^1^. Increasing evidence indicates that the phenotype and function of myeloid cells can be altered by the tumor conditions and converted into immunosuppressive cells^2^. Myeloid-derived suppressor cells (MDSCs) have been identified as an immature myeloid heterogeneous population and markedly expand and accumulate in the tumor environment^3–5^. Moreover, MDSCs exhibit a strong suppressive function towards T cell-mediated immune responses and can also facilitate tumor progression (e.g., tumor growth, angiogenesis, and metastasis)^6–8^. Thus, it is necessary to illuminate the molecules and mechanism associated with regulating the abnormal differentiation of hematopoietic progenitor cells into MDSCs.

In the tumor microenvironment, MDSCs are generated from multipotent hematopoietic progenitor cells following stimulation by cytokines and growth factors released by tumor and stromal cells^9–12^. Our recent study demonstrated a novel role of two chemokines, CXCL1 and CXCL2, in promoting monocytic MDSCs (mo-MDSCs) generation by favoring the differentiation of bone marrow cells under tumor conditions^13^. However, the precise precursors that CXCL1 and CXCL2 act upon and the related signaling pathways remain unknown. CXC chemokine receptor 2 (CXCR2) is the receptor for CXCL1 and CXCL2^14,15^. The crucial immune function of CXCR2 is to regulate neutrophil mobilization from the bone marrow to the blood and promote the migration of the neutrophil into inflammatory sites^16^. Moreover, CXCR2 has been shown to play a role in maintaining the fate of normal hematopoietic stem/progenitor cells, including survival and self-renewal^17^. CXCR2 also plays a significant role in melanoma growth, angiogenesis, and metastasis^18^. In addition, CXCR2-deficient mice display the characteristics of decreased Gr1^+^ tumor-associated granulocytes, F4/80^+^ tumor-associated macrophages, and CD11b^+^Gr1^+^ MDSCs, as well as delayed tumor progression^19^. Collectively, these studies suggest that CXCR2 might participate in MDSCs accumulation. MDSCs can be further divided into two subsets: (1) granulocytic MDSCs (G-MDSCs, CD11b^+^ Ly6G^+^ Ly6C^low^); and (2) mo-MDSCs (CD11b^+^ Ly6G^−^ Ly6C^hig^) in mice^20^. Although G-MDSCs account for the majority of MDSCs, mo-MDSCs have been found to be no less immunosuppressive than G-MDSCs on a per cell basis^21^. Importantly, neoplastic and tumor-associated stromal cells release multiple tumor-derived soluble factors that can increase mo-MDSCs expansion^1^. Nevertheless, the relevant mechanism by which CXCR2 regulates mo-MDSCs accumulation in the tumor microenvironment remains unexplored.

Here, we reported that granulocyte and macrophage progenitor cells (GMPs) express CXCR2, and CXCR2 deficiency reduces the ability of GMPs to give rise to macrophage and dendritic cell progenitor cells (MDPs), leading to the decreased mo-MDSCs generation. Furthermore, SAP18 expression was found to be up-regulated in hematopoietic stem cells and their progenitor cells (HSPCs) of CXCR2-deficient tumor-bearing mice. The increase of SAP18 expression inhibited the ERK/STAT3 signaling pathway, which regulates the differentiation of HSPCs into mo-MDSCs. Thus, these findings reveal a novel role for CXCR2 through which SAP18/ERK/STAT3 signaling regulates hematopoietic cells differentiation in the tumor microenvironment.

## Results

### Lack of CXCR2 in tumor-bearing mice leads to an attenuation of mo-MDSCs accumulation

**Fig. 1:**
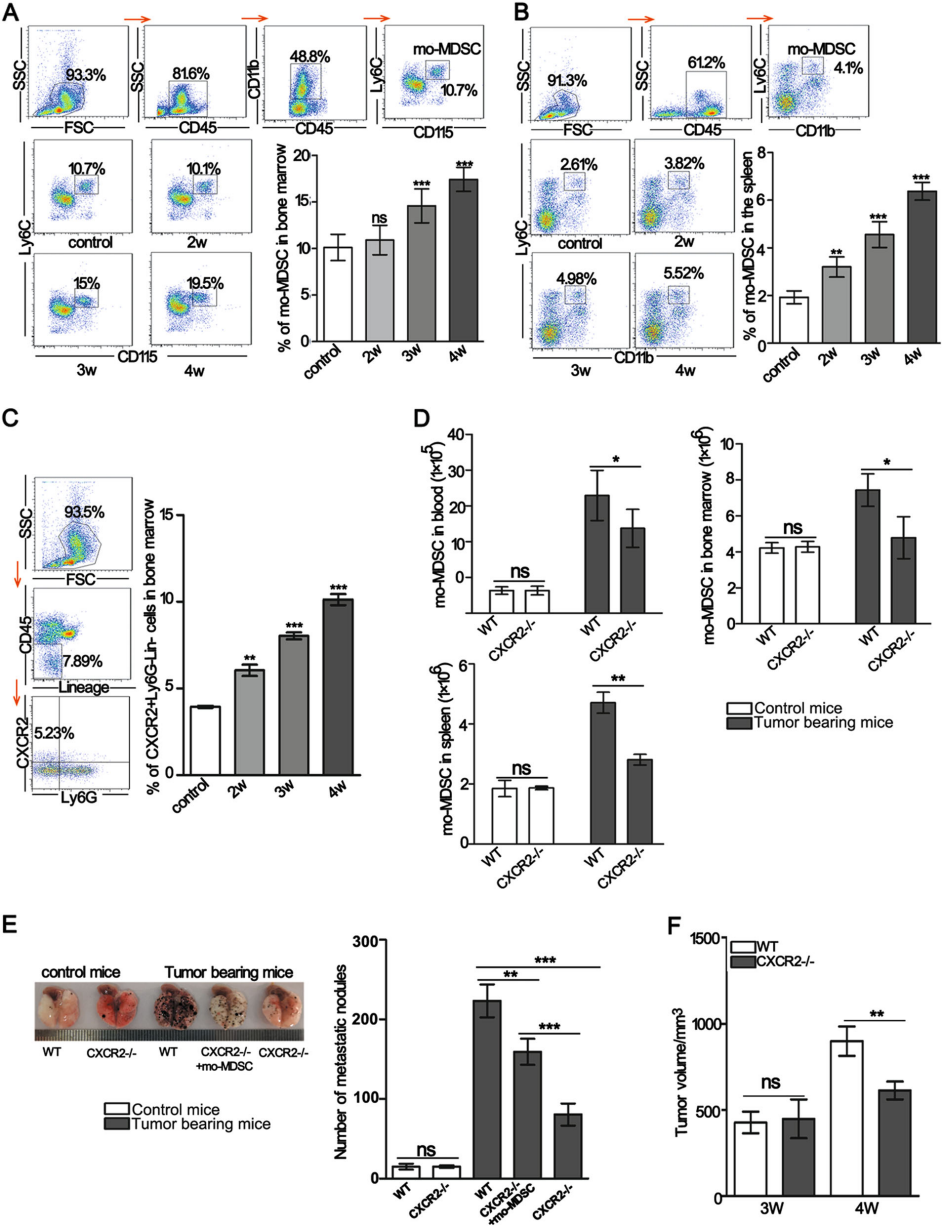
A lack of CXCR2 leads to reduced mo-MDSCs accumulation and delayed tumor progression. **a**, **b** The percentage of mo-MDSCs in the bone marrow and spleen of tumor-bearing mice was analyzed by flow cytometry. The samples were analyzed on the second, third, and week 4th of tumor-bearing. **c** The CXCR2 positive cells in bone marrow were analyzed by flow cytometry. The mice were treated as described in **a**, **b**. **d** Absolute number of mo-MDSCs in the blood, bone marrow, and spleen of control mice or mice bearing tumors after 3 weeks. **e** The number of metastatic foci was detected in the lungs of WT control mice, CXCR2−/− control mice, 2-week WT, 2-week CXCR2−/−, and adoptively transferred mo-MDSCs in two-week CXCR2−/− tumor-bearing mice. All mice were sacrificed at 2 weeks after a tail vein injection of B16F10 cells. The transferred mo-MDSCs were isolated from the blood of WT tumor-bearing mice. **f** B16F10 cells were subcutaneously inoculated into WT or CXCR2−/− mice, and the size of tumors was measured at the indicated time points. Bars represent the mean ± SD of five independent experiments. A one-way ANOVA with repeated measures followed by a Dunnett’s post hoc test or a two-way ANOVA followed by Holm-Sidak’s post hoc test were used to determine the level of statistical significance (*p < 0.05; **p < 0.01; and ***p < 0.001; ns, not significant)

Tumor progression is accompanied by mo-MDSCs accumulation^1,4^. First, the changes to mo-MDSCs in the bone marrow and spleen were examined in a B16F10-bearing mouse model. The data showed that the percentage of mo-MDSCs in the bone marrow was significantly increased in tumor-bearing mice after 3 weeks (Fig. 1a). The percentage of mo-MDSCs in the spleen was also increased with the extension of tumor-bearing time (Fig. 1b). Next, the expression of CXCR2 in bone marrow cells was analyzed. The results showed that the expression of CXCR2 on lineage^−^CD45^+^ Ly6G^−^ bone marrow cells increased with the extension of tumor-bearing time (Fig. 1c). To investigate whether the expression of CXCR2 was related to mo-MDSCs accumulation under tumor conditions, we analyzed the number of mo-MDSCs in the blood, bone marrow, and spleen of wild-type (WT) and CXCR2-deficient (CXCR2−/−) mice. The results showed that normal myelopoiesis was not affected in CXCR2−/− mice (Fig. S1A–D), however, the number of mo-MDSCs were all significantly reduced in CXCR2−/− mice bearing tumors after 3 weeks (Fig. 1d), indicating that CXCR2 was required for mo-MDSCs expansion under tumor conditions.

To determine whether the decreased number of mo-MDSCs resulting from a CXCR2 deficiency contributes to the suppression of tumor progression, B16F10 cells were intravenously injected into WT and CXCR2−/− mice. The results showed that the number of lung metastatic nodules was substantially reduced in CXCR2-/- tumor-bearing mice. However, when the mo-MDSCs were supplemented in CXCR2−/− tumor-bearing mice, the number of lung metastatic nodules was increased compared with the CXCR2−/− tumor-bearing mice (Fig. 1e). A significant inhibition in melanoma growth was observed in the CXCR2−/− mice compared with WT mice after bearing tumors for 3 weeks (Fig. 1f). These results suggest that a CXCR2 deficiency impairs the establishment of a tumor-supportive microenvironment by reducing mo-MDSCs accumulation.

### The differentiation of myeloid progenitor cells into mo-MDSCs is impaired in CXCR2−/− tumor-bearing mice

**Fig. 2:**
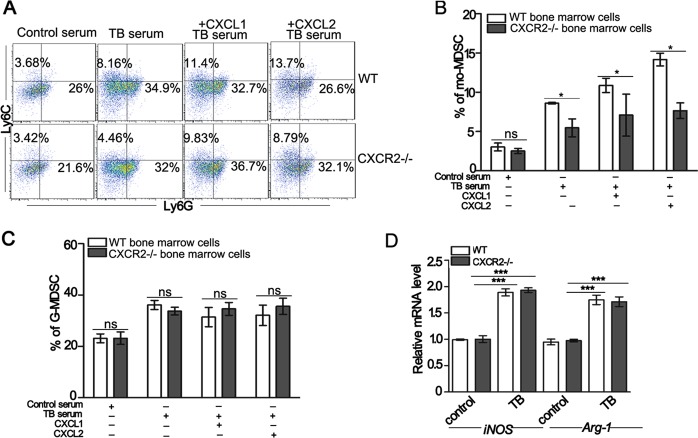
CXCR2 deficiency impairs the differentiation of myeloid progenitor cells into mo-MDSCs. **a** The percentage of mo-MDSCs and G-MDSCs induced from WT or CXCR2−/− bone marrow cells were detected by flow cytometry. The bone marrow cells were treated with the serum of control mice, tumor-bearing mice (TB), tumor-bearing mice + 20 ng/mL CXCL1, or tumor-bearing mice +20 ng/mL CXCL2 in the presence of GM-CSF for 5 days in vitro. **b**, **c** Quantitative analyses of mo-MDSCs and G-MDSCs as shown in **a**. **d** The relative level of *Arg1 and iNOS* mRNA expression in mo-MDSCs was analyzed by qPCR. The mo-MDSCs were sorted from WT or CXCR2−/− bone marrow progenitor cells induced by the serum of control (control) or tumor-bearing mice (TB) for 5 days. The bars represent the mean ± SD of five independent experiments. A one-way ANOVA with repeated measures followed by a Dunnett’s post hoc test or two-way ANOVA followed by a Holm-Sidak’s post hoc test show the level of statistical significance (*p < 0.05; **p < 0.01; and ***p < 0.001; ns, not significant)

To determine whether CXCR2 contributes to mo-MDSCs expansion, bone marrow progenitor cells from WT or CXCR2−/− mice were exposed to the serum isolated from different mice. The results showed that the serum of tumor-bearing mice exhibited a substantially increased proportion of mo-MDSCs than that of control mice and the addition of CXCL1 or CXCL2 induced a greater proportion of mo-MDSCs compared to the serum of only tumor-bearing mice. Furthermore, the differentiation of CXCR2−/− bone marrow progenitor cells into mo-MDSCs was markedly reduced compared with the WT mice in the serum of tumor-bearing mice or tumor-bearing mice supplemented with recombinant CXCL1 or CXCL2 (Fig. 2a, b). There were no differences in the proportion of G-MDSCs between the bone marrow progenitor cells of WT and CXCR2−/− mice (Fig. 2c). These results suggest that CXCR2 could drive mo-MDSCs accumulation. Next the mo-MDSCs were sorted from the groups exposed to the serum of control or tumor-bearing mice, and the level of arginase-1 (*Arg-1*) and inducible nitric oxide synthase (*iNOS*) mRNA was detected. The results showed that there were no differences between the level of *Arg-1* or *iNOS* mRNA in mo-MDSCs induced from WT and CXCR2−/− mice (Fig. 2d). There was also no significant difference in the proliferation and apoptosis of mo-MDSCs in WT and CXCR2−/− mice (Fig. S2A–D). And mo-MDSCs from CXCR2−/− mice gave rise to a similar percentage of CD11b^+^CD11c^+^ or CD11b^+^F4/80^+^ cells as the mo-MDSCs from WT mice upon appropriate stimulation^22^ (Fig. S2E and F). Taken together, these results indicate that the impaired differentiation of hematopoietic progenitor cells into mo-MDSCs is responsible for the decreased mo-MDSCs accumulation in CXCR2−/− tumor-bearing mice.

### MDPs, the hematopoietic progenitor cells of mo-MDSCs, are decreased in CXCR2−/− mice

**Fig. 3:**
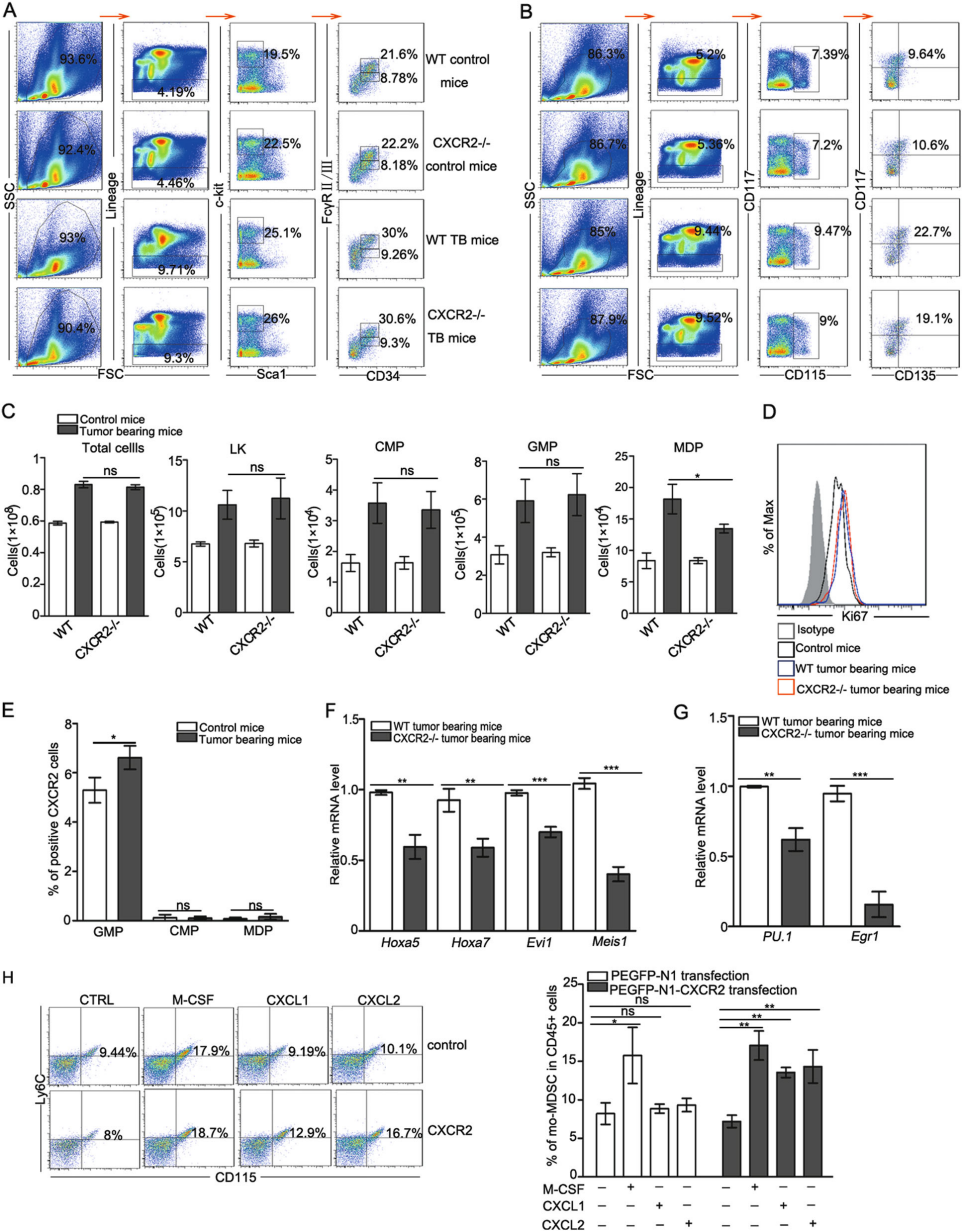
Lack of CXCR2 decreases the production of MDPs, the hematopoietic progenitors of mo-MDSCs. **a**, **b** The classification of HSPCs was analyzed by flow cytometry. Bone marrow cells from control and tumor-bearing mice were stained with antibodies specific to Lin-specific antigens, as well as Sca-1, c-Kit (CD117), CD34, FcγRII/III, CD115, and CD135. **c** The total number of bone marrow cells and quantitative analyses of HSPCs subsets as shown in **a**, **b**: LKs (Lin^−^Sca-1^−^c-Ki^+^), CMPs (LK, FcγRII/III^int^CD34^+^), GMPs (LK, FcγRII/III^hi^CD34^+^), MDPs (Lin^−^c-Kit^+^CD135^+^CD115^+^ Ly6C^−^CD11b^−^). **d** The expression of Ki67 in MDPs from WT control mice, WT, and CXCR2−/− tumor-bearing mice was analyzed by flow cytometry. **e** The expression of CXCR2 in GMPs, CMPs, and MDPs was analyzed by flow cytometry in control and tumor-bearing mice. **f**, **g** The level of *Hoxa5, Hoxa7, Evi1, Meis1, Pu.1*, and *Egr1* mRNA expression in the HSPCs of WT and CXCR2−/− tumor-bearing mice was evaluated by qPCR. **h** The percentage of mo-MDSCs was analyzed by flow cytometry in 32D clone three cells transfected with an empty vector or CXCR2. The treated cells were administered with M-CSF, CXCL1, or CXCL2 for 5 days in the presence of GM-CSF. The 32D clone three cells transfected with CXCR2 were treated with an equal concentration of M-CSF as the positive control group. Bars represent the mean ± SD of five independent experiments. A one-way ANOVA with repeated measures followed by a Dunnett’s post hoc test or two-way ANOVA followed by a Holm-Sidak’s post hoc test was used to determine the level of statistical significance (*p < 0.05; **p < 0.01; and ***p < 0.001; ns, not significant)

Hematopoietic stem cells (HSCs) undergo a cascade of multiple progenitor cell stages to generate all the blood cell lineages required for an immune response^23^. Common myeloid progenitor cells (CMPs) give rise to GMPs, which generate MDPs, and MDPs give rise to mo-MDSCs within the tumor environment^4^. We next examined whether CXCR2 deficiency changed the number of progenitor cells in a specific stage required for mo-MDSCs differentiation, thereby leading to the decreased mo-MDSCs generation. To this end, the number of total bone marrow cells, LKs (Lin^−^Sca-1^−^c-Kit^+^), CMPs (LK, FcγRII/III^int^CD34^+^), GMPs (LK, FcγRII/III^hi^ CD34^+^), and MDPs (Lin^-^CD117^+^CD115^+^CD135^+^Ly6C^−^CD11b^−^) were examined (Fig. 3a, b). There was no difference in the number of total bone marrow cells, LKs, CMPs, and GMPs between the WT and CXCR2 tumor-bearing mice, however, the number of MDPs of CXCR2−/− tumor-bearing mice was remarkably reduced (Fig. 3c). The results suggest that a CXCR2 deficiency leads to perturbations in the HSPC compartment. Next, the proliferative activity of MDPs was assessed. The results showed that the proliferative activity of MDPs was similar between WT and CXCR2−/− tumor-bearing mice (Fig. 3d), suggesting that the CXCR2 deficiency did not impair the proliferative activity of MDPs. We next examined the expression of CXCR2 on CMPs, GMPs, and MDPs. GMPs expressed remarkably CXCR2, but CMPs and MDPs did not express CXCR2 (Fig. 3e), suggesting that the CXCR2 deficiency influences the differentiation of GMPs into MDPs, which leads to a decrease in mo-MDSCs. The relative level of hematopoiesis gene (e.g., *Hoxa5, Hoxa7, Evi1*, and *Meis1*^24,25^) mRNA expression was decreased in CXCR2−/− tumor-bearing mice (Fig. 3f). And the important transcription factors for the differentiation of monocytes (e.g., *Pu.1* and *Egr1*) were expressed at low levels in CXCR2−/− tumor-bearing mice (Fig. 3g). These data suggest that a deficiency in CXCR2 reduces the hematopoietic activity in tumor conditions. To confirm the role of CXCR2 on the differentiation of GMPs to MDPs, CXCR2 was transfected into 32D clone three cells to simulate GMPs (Fig. S3A and B) and recombinant CXCL1 or CXCL2 was administered to the treated 32D clone three cells. The data showed that 32D clone three cells transfected with CXCR2 generated more mo-MDSCs in the presence of M-CSF, CXCL1, or CXCL2 (Fig. 3h). This suggests that CXCR2 activated by CXCL1 or CXCL2 promotes GMPs to generate MDPs, which give rise to mo-MDSCs. Taken together, these results indicate that the lack of CXCR2 on GMPs reduces the generation of MDPs, which are the hematopoietic progenitor cells of mo-MDSCs under tumor conditions.

### CXCR2 deficiency reduces the differentiation of GMPs to mo-MDSCs through suppression of the ERK/STAT3 pathway

**Fig. 4:**
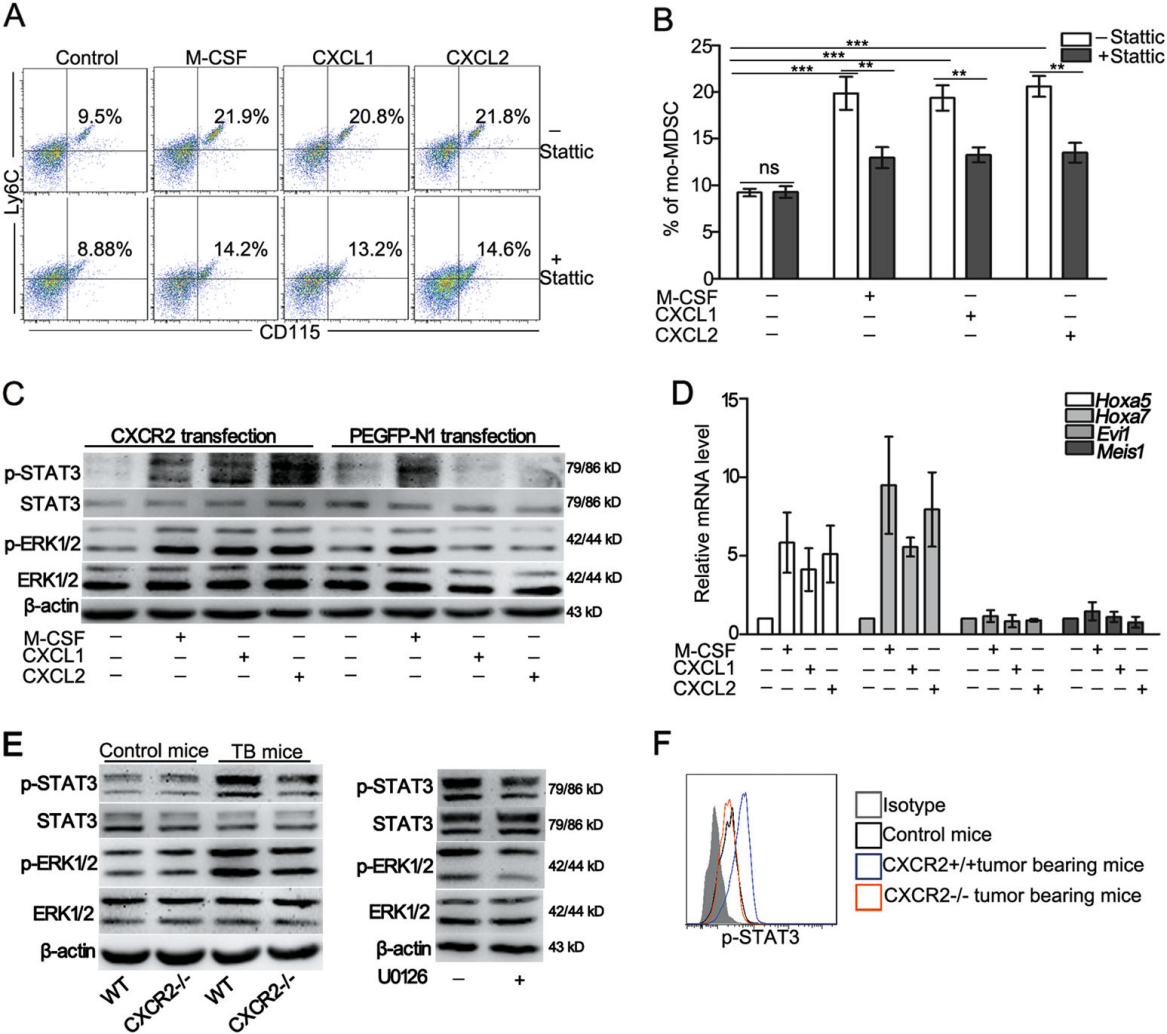
CXCR2 deficiency reduces the differentiation of GMPs into mo-MDSCs by suppressing the ERK/STAT3 pathway. **a** The percentage of mo-MDSCs was analyzed by flow cytometry after the administration of Stattic (the inhibitor of STAT3) to 32D clone three cells transfected with CXCR2 for 5 days. The treated cells were incubated with M-CSF, CXCL1, or CXCL2 in the presence of GM-CSF. **b** Quantitative analyses of the data presented in **a**. **c** Analysis of the expression of STAT3, p-STAT3, ERK1/2, and p-ERK1/2 in the 32D clone three cells transfected with an empty vector or CXCR2 and stimulated with M-CSF, CXCL1, or CXCL2 for 4 h. **d** The level of *Hoxa5, Hoxa7, Evi1*, and *Meis1* mRNA was evaluated by qPCR in the 32D clone three cells that were treated as described in **c**. **e** The expression of ERK1/2, p-ERK1/2, STAT3, p-STAT3, and β-actin in HSPCs was analyzed by Western blot. Left: HSPCs were sorted from tumor-bearing mice (TB) or control mice; Right: HSPCs isolated from tumor-bearing mice that were tail vein injected with and without U0126 (inhibitor of ERK1/2). **f** The level of phosphorylated STAT3 was analyzed by flow cytometry in the GMPs of tumor-bearing or control mice. Bars represent the mean ± SD of five independent experiments. A one-way ANOVA with repeated measures followed by Dunnett’s post hoc test or a two-way ANOVA followed by a Holm-Sidak’s post hoc test was used to determine the level of statistical significance (*p < 0.05; **p < 0.01; and ***p < 0.001; ns, not significant)

Cytokines and growth factors secreted by tumor and stromal cells regulate the differentiation of myeloid progenitor cells through a variety of transcription factors, of which the STAT3 plays a critical role^3,26^. Thus, the effect of STAT3 on the differentiation of GMPs into mo-MDSCs was examined. The results showed that treatment with the STAT3 inhibitor, Stattic, resulted in an significant decrease in the inductive effect of cytokines on mo-MDSCs accumulation (Fig. 4a, b), suggesting that STAT3 is involved in activated CXCR2 pathways for regulating mo-MDSCs accumulation. To specify the role of CXCR2 associated with the signal transduction in the activation of STAT3, 32D clone three cells were transfected with either CXCR2 or an empty vector and incubated with M-CSF, CXCL1, or CXCL2. The results showed that treatment with M-CSF increased the phosphorylation of ERK1/2 and STAT3 in the 32D clone three cells transfected with either CXCR2 or an empty vector. However, treatment with CXCL1 or CXCL2 only activated ERK1/2 and STAT3 in the 32D clone three cells transfected with CXCR2 (Fig. 4c). To verify whether CXCR2 drives the hematopoietic activity of 32D clone three cells, the level of *Hoxa5, Hoxa7, Evi1*, and *Meis1* mRNA was assessed. The results show that *Hoxa5* and *Hoxa7* were increased in the 32D clone three cells transfected with CXCR2 and exposed to M-CSF, CXCL1, or CXCL2 (Fig. 4d). These data suggest a vital role of the ERK/STAT3 axis in the CXCR2-mediated regulation of mo-MDSCs accumulation.

The phosphorylation of ERK1/2 and STAT3 was detected in HSPCs isolated from different mice. The results showed that the phosphorylation of ERK1/2 and STAT3 was up-regulated, whereas a lack of CXCR2 suppressed the activation of ERK1/2 and STAT3 in the tumor conditions (Fig. 4e). Treatment with the ERK1/2 inhibitor, U0126, dramatically inhibited ERK1/2 phosphorylation, and STAT3 phosphorylation was also decreased (Fig. 4e). Other molecules (e.g., PARP1, JAK2, and SHP2) related to STAT3 activation ^27-29^ were also examined, and the results showed that the signaling molecules were not substantially influenced by a CXCR2 deficiency (Fig. S4). Furthermore, STAT3 phosphorylation was also decreased in the GMPs of CXCR2-/- tumor-bearing mice (Fig. 4f). These data further verify that CXCR2 regulates the differentiation of GMPs into mo-MDSCs through the ERK/STAT3 pathway.

### SAP18 regulated by CXCR2 under tumor conditions is critical for the generation of mo-MDSCs

**Fig. 5:**
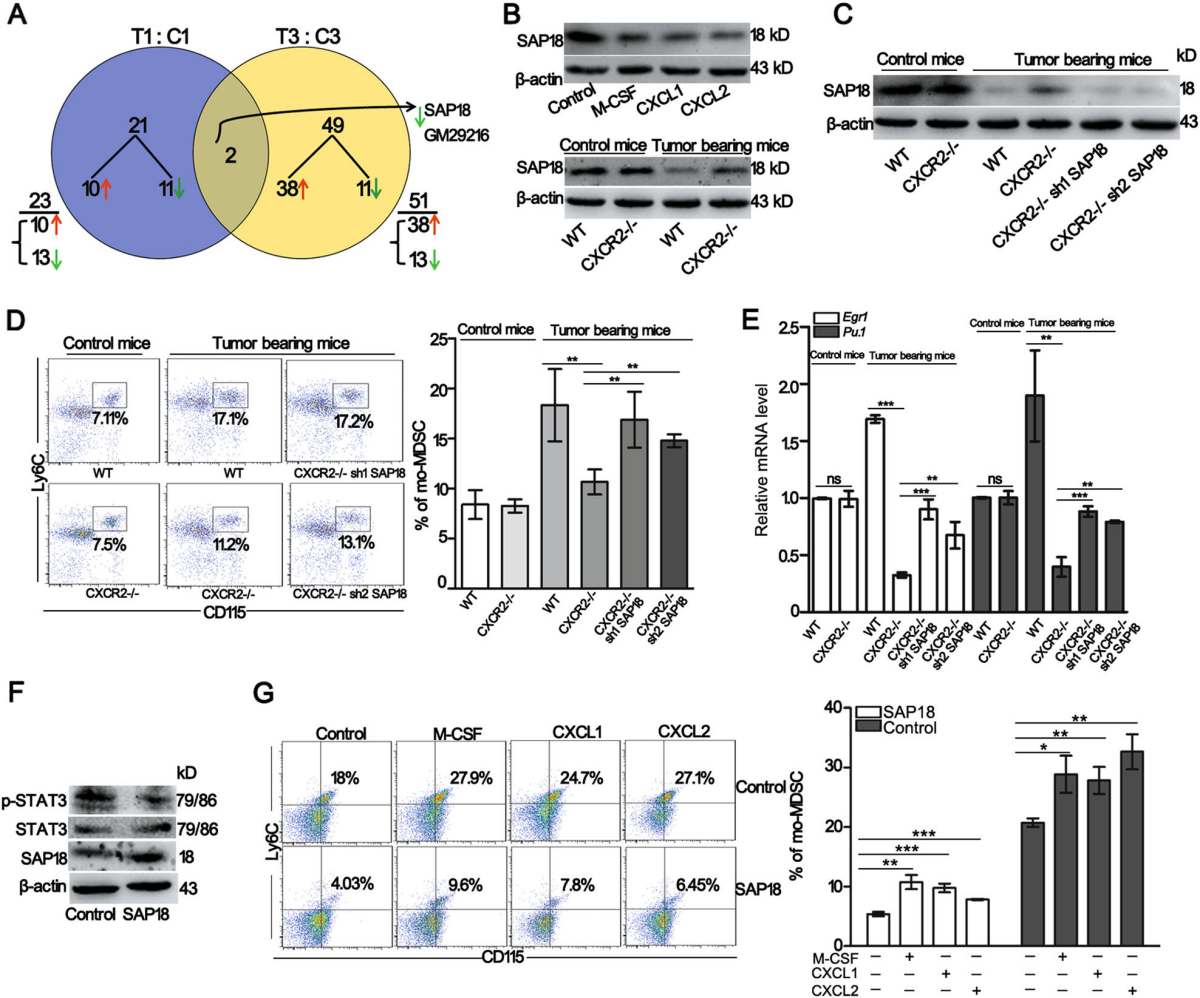
SAP18 regulated by CXCR2 under tumor conditions is critical for the generation of mo-MDSCs. **a** Venn diagrams show the genes that are consistently 1.5-fold upregulated or two-fold downregulated in 32D clone three cells transfected with CXCR2 or the empty vector, and both cells were incubated with CXCL1 and CXCL2 for 4 h. The total amount of differentially expressed genes within a given subset is reported as an underlined number. Below this number (next to a brace), its composition in terms of upregulated (↑, red) or downregulated (↓, green) genes is indicated. The genes at the intersection that are systematically either upregulated or downregulated are termed “coherent”. The number of coherent genes within the intersection was noted, and their identities (gene names) are reported at the right end of the diagrams. **b** The expression of SAP18 was analyzed by Western blot in 32D clone three cells transfected with CXCR2 and administrated with M-CSF, CXCL1, or CXCL2 for 4 h (top). The expression of SAP18 in HSPCs of tumor-bearing or control mice was analyzed by western blot (down). **c** The expression of SAP18 in HSPCs was evaluated in control and tumor-bearing mice. **d** The percentage of mo-MDSCs in the bone marrow was analyzed by flow cytometry. The mice were treated as described in **c**. **e** The level of *Pu.1* and *Egr1* mRNA in the HSPCs was detected by qPCR. The mice were treated as described in **c**. **f** The expression of STAT3, p-STAT3, and SAP18 was tested in 32D clone three cells transfected with CXCR2 (control) or 32D clone three cells transfected with CXCR2 and SAP18 (SAP18). All of the cells were treated with M-CSF, CXCL1, or CXCL2 for 4 h. **g** The percentage of mo-MDSCs was analyzed by flow cytometry. The cells were treated as described in **f**. All of the cells were incubated with M-CSF, CXCL1 or CXCL2 for 5 days in the presence of GM-CSF. Bars represent the mean ± SD of five independent experiments. A one-way ANOVA with repeated measures followed by a Dunnett’s post hoc test or a two-way ANOVA followed by a Holm-Sidak’s post hoc test were used to evaluate the level of statistical significance (*p < 0.05; **p < 0.01; and ***p < 0.001; ns, not significant)

The ERK1/2 phosphorylation induced by treatment with isoproterenol was insufficient to increase the ERK1/2 phosphorylation and mo-MDSCs generation in CXCR2−/− tumor-bearing mice as shown in WT tumor-bearing mice (Fig. S5A−D). To thoroughly investigate whether CXCR2 signaling in HSPCs activates other molecules involved in the differentiation of HSPCs into mo-MDSCs, 32D clone three cells transfected with CXCR2 or an empty vector were incubated with CXCL1 and CXCL2, and RNA sequencing was performed. A venn diagram analysis of the predicted CXCR2 targets was performed from the two independent databases and the data revealed two candidate targets of CXCR2: SAP18 and Gm29216 (Fig. 5a). Since Gm29216 is an unprocessed pseudogene, we hypothesized that SAP18 was the candidate target of CXCR2. Immunoblot analyses showed that SAP18 expression was decreased in 32D clone three cells transfected with CXCR2 and incubated with M-CSF, CXCL1, or CXCL2 for 4 h (Fig. 5b top). Next, SAP18 expression was analyzed in HSPCs. The results showed that SAP18 expression was decreased in the HSPCs of WT tumor-bearing mice; however, SAP18 expression was increased in CXCR2−/− tumor-bearing mice (Fig. 5b down). These observations suggest that CXCR2 could regulates SAP18 expression under tumor conditions.

Based on a previous report^30^, lentivirus-based RNA interference against SAP18 was intrapleurally injected into CXCR2−/− mice bearing tumors for 3 weeks to test the function of SAP18 in the differentiation of HSPCs into mo-MDSCs. Immunoblot analyses showed that the knock down of SAP18 was evident in the HSPCs of CXCR2−/− tumor-bearing mice (Fig. 5c). Next, the percentage of mo-MDSCs in the bone marrow was examined. The results showed that knocking down SAP18 in CXCR2−/− tumor-bearing mice increased the percentage of mo-MDSCs (Fig. 5d), and the level of *Pu.1* and *Egr1* mRNA was also recovered (Fig. 5e). These data suggest that SAP18 negatively regulates mo-MDSCs generation. To verify the effect of SAP18 on mo-MDSCs accumulation, 32D clone three cells transfected with CXCR2 and over-expressing SAP18 were incubated with M-CSF, CXCL1, or CXCL2. The results showed that SAP18 overexpression in 32D clone three cells transfected with CXCR2 inhibited STAT3 phosphorylation and decreased mo-MDSCs accumulation (Fig. 5f, g). Moreover, SAP18 overexpression in 32D clone three cells transfected with CXCR2 also inhibited the level of *Pu.1*, and *Egr1* mRNA (Fig. S6). These data suggest that activated CXCR2 on HSPCs increases mo-MDSCs generation by inhibiting SAP18 expression under tumor conditions.

### Transcriptional suppression of PI3Kγ and HRAS by SAP18 attenuates ERK-mediated STAT3 activation and leads to an inhibition of mo-MDSCs accumulation

**Fig. 6:**
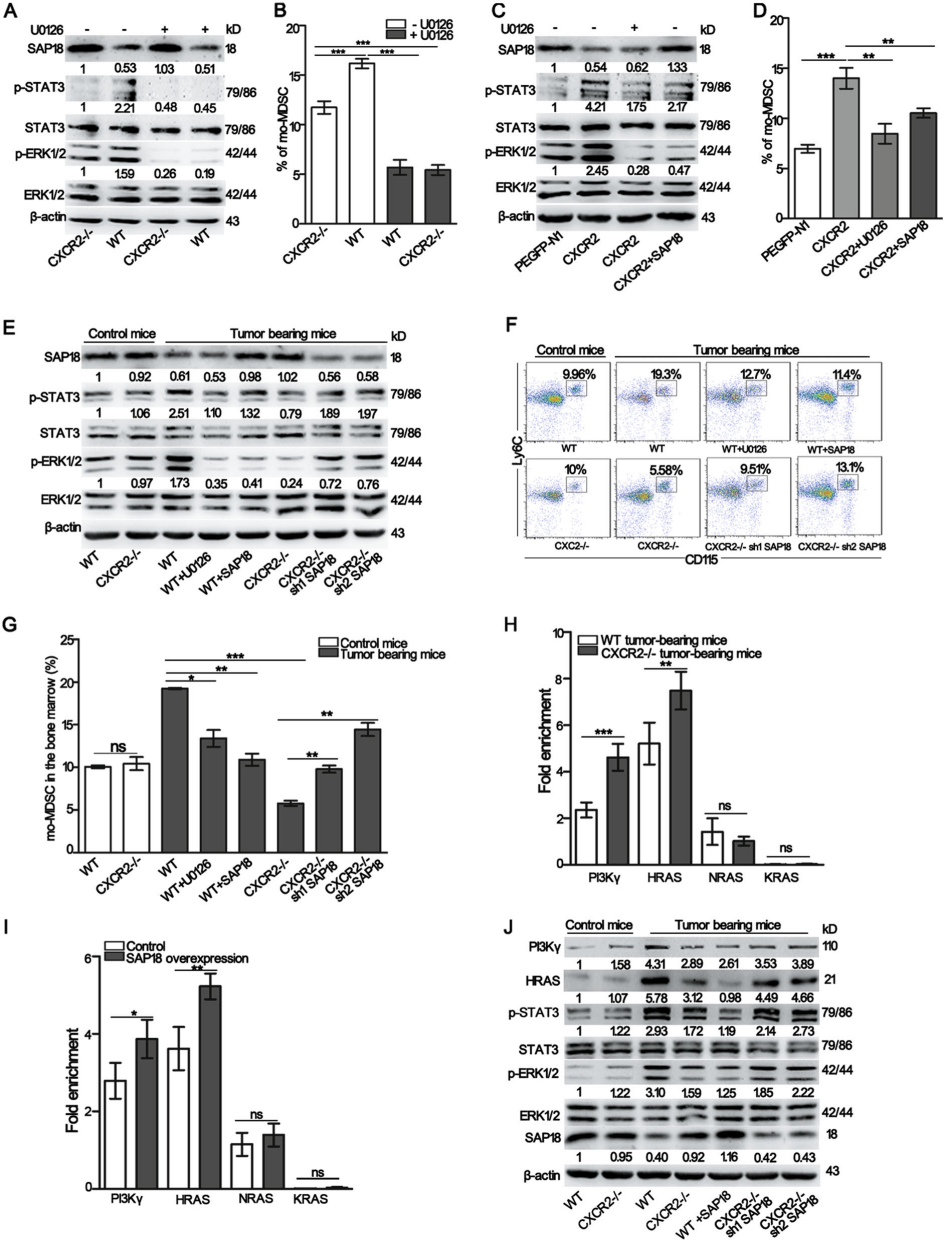
The transcriptional suppression of PI3Kγ and HRAS regulated by SAP18 attenuates ERK-mediated STAT3 activation and leads to an inhibition of mo-MDSCs accumulation. **a** The expression of ERK1/2, p-ERK1/2, STAT3, p-STAT3, and SAP18 in the HSPCs isolated from WT, CXCR2−/−, U0126-treated WT, and U0126-treated CXCR2−/− tumor-bearing mice was determined by western blot. **b** The mice were treated as described in **a** and the percentage of mo-MDSCs in the bone marrow was analyzed by flow cytometry. **c** The expression of ERK1/2, p-ERK1/2, STAT3, p-STAT3, and SAP18 in the 32D clone three cells transfected with the empty vector, CXCR2, CXCR2 administrated with U0126 or CXCR2 combined with SAP18. All cells were incubated with CXCL1 and CXCL2 for 4 h. **d** The cells were treated as described in **c** and cultured with CXCL1 and CXCL2 for 5 days in the presence of GM-CSF. The percentage of mo-MDSCs was evaluated from 32D clone three cells by flow cytometry. **e** The expression of ERK1/2, p-ERK1/2, STAT3, p-STAT3, and SAP18 was analyzed by Western blot in the HSPCs isolated from WT control mice, CXCR2−/− control mice, WT, SAP18 over-expressing WT, U0126-treated WT, CXCR2−/−, and shSAP18 CXCR2−/− tumor-bearing mice. **f** The mice were treated as described in **e** and the percentage of mo-MDSCs was analyzed in the bone marrow by flow cytometry. **g** Quantitative analyses of the data described in **f**. **h** ChIP analysis using anti-SAP18 antibodies in HSPCs. HSPCs were sorted from both WT and CXCR2−/− tumor-bearing mice. **i** ChIP analysis using an anti-mSinA antibody in HSPCs. HSPCs were sorted from WT and SAP18 over-expressing WT tumor-bearing mice. **j** The expression of HRAS, PI3Kγ, ERK1/2, p-ERK1/2, STAT3, p-STAT3, and SAP18 in the HSPCs was analyzed by Western blot. HSPCs were sorted from WT control mice, CXCR2−/− control mice, WT, CXCR2−/−, SAP18 over-expressing WT and shSAP18 CXCR2−/− tumor-bearing mice. Quantitative analyses of the protein in Fig. 6 were conducted by Image J software. SAP18, HRAS and PI3Kγ were normalized over β-actin. p-ERK1/2 was normalized over ERK1/2, and p-STAT3 was normalized over STAT3. Bars represent the mean ± SD of five independent experiments. A one-way ANOVA with repeated measures followed by a Dunnett’s post hoc test or a two-way ANOVA followed by a Holm-Sidak’s post hoc test were used to evaluate the level of statistical significance (*p < 0.05; **p < 0.01; and ***p < 0.001; ns, not significant)

Based on the above findings, we assumed that there was a relationship between ERK1/2 and SAP18. To test this hypothesis, WT and CXCR2−/− tumor-bearing mice were intravenously injected with U0126. The results showed that the inhibition of ERK1/2 phosphorylation had no effect on SAP18 expression in HSPCs (Fig. 6a and Fig. S7A), but decreased the proportion of mo-MDSCs (Fig. 6b). Next, 32D clone three cells transfected with CXCR2 were treated with U0126 or over-expressed SAP18. Immunoblot analyses showed that SAP18 overexpression inhibited ERK1/2 phosphorylation (Fig. 6c and Fig. S7B), which exhibited an effect similar to that of the ERK1/2 inhibitor on STAT3 phosphorylation and mo-MDSCs generation (Fig. 6c, d). These results indicate that SAP18 is responsible for ERK1/2 activation. To specify the role of SAP18 on ERK1/2 activation, a lentivirus-based SAP18 overexpression sequence was intrapleurally injected into WT tumor-bearing mice to overexpress SAP18, and lentivirus-based RNA interference against SAP18 was intrapleurally injected into CXCR2−/− tumor-bearing mice to knock down SAP18. Immunoblot analyses showed that ERK1/2 phosphorylation was markedly decreased in the WT tumor-bearing mice over-expressing SAP18 and increased in CXCR2−/− tumor-bearing mice treated with the SAP18 knock down (Fig. 6e and Fig. S7C). Moreover, the percentage of mo-MDSCs in the bone marrow was decreased by up-regulating SAP18 expression and increased by knocking down SAP18 (Fig. 6f, g). Consistently, there were similar changes in the percentage of mo-MDSCs in the blood (Fig. S8A and B); and, SAP18 did not influence the percentage of G-MDSCs in the blood (Fig. S8A and C). These data suggest that activated CXCR2 regulates ERK-mediated STAT3 activation by restraining SAP18 expression under tumor conditions.

GTP-bound RAS (including HRAS, KRAS, and NRAS) activates the ERK1/2 signaling cascade, and PI3Kγ plays a key role in sustaining the function of ERK during multiple cellular processes^31,32^. Thus, we next performed ChIP-qPCR analyses to investigate the mechanisms by which SAP18 regulates ERK1/2 activation. The results showed that treatment with the anti-SAP18 antibodies (Abs) enriched the DNA fragment at the HRAS and PI3Kγ promoters. Moreover, enrichment with the anti-SAP18 Abs was enhanced in CXCR2−/− tumor-bearing mice (Fig. 6h). Since SAP18 has been identified as a subunit of the transcriptional co-repressor, Sin3A-HDAC complex^33^, we next investigated whether mSin3A binds to the regulatory sequences of HRAS, KRAS, NRAS, or PI3Kγ. Treatment with anti-mSin3A Abs enriched the same DNA fragments which had been enriched by the anti-SAP18 Abs, and the enrichment by the anti-mSin3A Abs was enhanced when SAP18 was overexpressed in WT tumor-bearing mice (Fig. 6i). These results indicate that SAP18 binds to the regulatory sequences of HRAS and PI3Kγ, which enhances the binding of mSin3A to the promoter of HRAS and PI3Kγ to inhibit the transcription of HRAS and PI3Kγ. Immunoblot analyses further showed that the knockdown of SAP18 in CXCR2−/− tumor-bearing mice resulted in a substantial increase in HRAS and PI3Kγ expression. In addition, SAP18 overexpression in WT tumor-bearing mice markedly decreased the expression of HRAS and PI3Kγ and the expression of HRAS and PI3Kγ was closely connected with ERK1/2 activation (Fig. 6j and Fig. S7D). These results indicate that SAP18 participates in the activation of the ERK signaling pathway by inhibiting HRAS and PI3Kγ expression in HSPCs.

## Discussion

CXCR2 is typically described as a chemokine receptor expressed on neutrophils and plays a critical role in the regulation of neutrophil homeostasis^16,34^. CXCR2 is also expressed on microvascular endothelial cells and is responsible for mediating angiogenesis^35^. In the central nervous system, the interaction of CXCL1 and CXCR2 on oligodendrocyte progenitor cells prevents the apoptosis of these cells^36,37^. Moreover, CXCR2 plays a critical role in angiogenesis, tumorigenesis, and metastasis of cancer^38–41^. Recently, CXCR2 has been reported to play an important role in HSCs maintenance^17^. In the present study, we demonstrated that activated CXCR2 on HSPCs contributes to mo-MDSCs generation under tumor conditions. CXCR2 expressed on GMPs (Fig. 3e) and facilitates the differentiation of GMPs into MDPs (Fig. 3c), which results in mo-MDSCs generation (Fig. 1d). Other than a classical chemokine receptor, we found a novel function of CXCR2 in regulating the differentiation of hematopoietic progenitor cells under tumor conditions. The role of CXCR2 in cancer-related MDSCs expansion has been already described in preclinical models, which showed that CXCR2 signaling in the Ly6G^+^ MDSCs promotes pancreatic tumorigenesis and is required for pancreatic cancer metastasis^42^. The results we presented here suggest that CXCR2 could play a cell-type-specific role in mo-MDSCs generation. The expansion of mo-MDSCs is a critical step associated with immunosuppression and multiple CXCR2 antagonists exist. Thus, our findings identify CXCR2 as a potential pharmaceutical target for the inhibition of tumor progression.

It has been reported that the STAT3-mediated upregulation of S100A8, S100A9 and C/EBPβ in myeloid progenitors promotes MDSCs accumulation in cancer^43^. Moreover, STAT3 inhibits the differentiation of myeloid cells by downregulating IRF8 expression^44^. While the classic model of mo-MDSCs expansion only demonstrates that STAT3 regulates mo-MDSCs expansion, we have further verified that GMPs are the critical myeloid progenitor cells, of which STAT3 activity was increased during tumor-bearing conditions (Fig. 4f). The identification of GMPs is significant to elucidate the mechanisms of mo-MDSCs accumulation in the tumor microenvironment. Thus, our study contributes to the further understanding of the differentiation of myeloid progenitor cells into mo-MDSCs.

According to a previous report^45^, the concentration of STAT3 inhibitor, Stattic, used in this study was 3 μM, at which STAT3 inhibition is only partial (Fig. S9). The result suggests that other pathways might be involved in mo-MDSCs generation. It has been reported that Notch signaling and EZH2 pathway are involved in MDSCs generation ^46,47^, and HMGB1 facilitates mo-MDSCs generation via p38/NFκB/Erk1/2 pathway^5^. Therefore, further research will be needed to thoroughly clarify the mechanism of mo-MDSCs generation.

Our work also reports the novel discovery that SAP18 is an important molecule required for the coupling of CXCR2 to its downstream signaling molecules to modulate mo-MDSCs expansion. It is important to note that SAP18 was originally identified as a subunit of a transcriptional co-repressor (the Sin3A-HDAC complex) which mediates the interaction between Sin3A and DNA-bound transcription factors^33,48,49^. Our results showed that SAP18 expression was not affected by a CXCR2 deficiency in normal mice; however, SAP18 expression was increased when CXCR2 was deficient under tumor conditions (Fig. 5b). Thus, these data reveal a novel function for SAP18 in limiting mo-MDSCs expansion. Furthermore, we found that there was a reverse trend of ERK-mediated STAT3 activation in response to the alteration of SAP18 expression under tumor conditions. (Fig. 6e). ChIP studies indicated the direct binding of SAP18 at the regulatory sequences of HRAS and PI3Kγ and SAP18 overexpression enhanced the binding of mSin3A to the same regulatory sequences (Fig. 6h, i). These findings indicate that SAP18 inhibits HRAS and PI3Kγ expression by enhancing the binding of mSin3A to these regulatory sequences. It has been previously reported that ERK1/2 is activated via the RAS-regulated RAF-MEK1/2-ERK1/2 signaling pathway^50^. PI3Kγ also sustains the function of ERK in multiple cellular processes^32^. Our present findings show that SAP18 represents a novel upstream component of the ERK1/2 pathway.

**Fig. 7:**
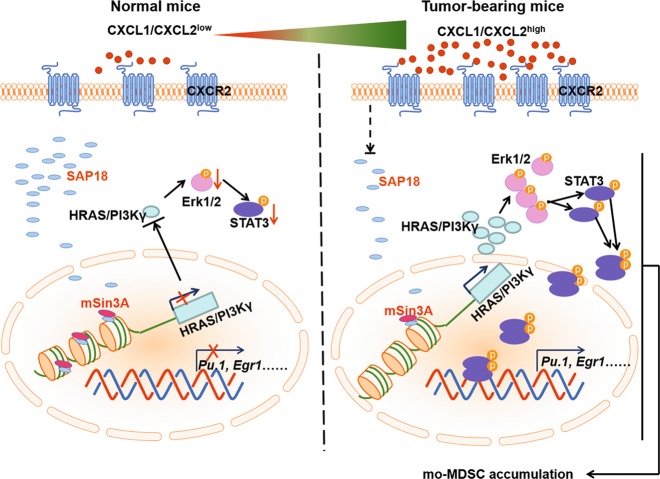
Schematic illustration of the proposed model. The level of CXCL1 and CXCL2 was very low under normal physiological conditions, but the level was greatly increased under tumor conditions. The increased CXCL1 and CXCL2 under tumor conditions binds to its receptor CXCR2 on GMPs. In this case, the GMPs expressed low level of SAP18 resulting in decreasing the binding of mSin3A to the promoter of HRAS and PI3Kγ, which enhanced the transcription of HRAS and PI3Kγ in the GMPs. The increased expression of HRAS and PI3Kγ promoted the phosphorylation of ERK1/2 and subsequently activated STAT3 signaling pathway to promote mo-MDSCs accumulation

In summary, we report CXCR2 as a novel contributor involved in the differentiation of HSPCs into mo-MDSCs. The increased binds of CXCL1 or CXCL2 to CXCR2 leads to reduced SAP18 expression under tumor conditions, which subsequently activates the ERK/STAT3 signaling pathway to promote mo-MDSCs accumulation (Fig. 7). The identification of the specific signaling pathway activated by CXCR2 provides a novel regulatory mechanism of STAT3 activation in myeloid cell differentiation and a potential strategy for reducing immune suppression in the tumor microenvironment.

## Methods and materials

### Mice and cell lines

Female C57BL/6J mice (8–10-weeks-old) were purchased from the Animal Experimental Center of Jilin University (Changchun, China) to be used as WT controls. CXCR2-deficient mice on a C57BL/6J background were provided by Dr. Hong Zhou (Department of Immunology, Nanjing Medical University). All mice were housed in a specific pathogen-free environment under protocols approved by the Animal Care Committee of Northeast Normal University, China, and all experiments related to the mice were performed in accordance with the approved guidelines. B16F10 cells and HEK-293T cells were maintained in Dulbecco’s Modified Eagle’s Medium (Gibco) supplemented with 10% heat-inactivated fetal bovine serum (Corning) and 1% penicillin/streptomycin. 32D clone three cells were maintained in RPMI 1640 medium supplemented with 10% heat-inactivated fetal bovine serum and 10% mouse interleukin (IL)-3 (213–13, Peprotech) culture supplement.

### In vitro differentiation experiments

Bone marrow cells were obtained from the femurs of mice^51^. The cells were ground and filtered through a 200-μm cell strainer and erythrocytes were eliminated using hypotonic lysis buffer (155 mM NH_4_Cl, 0.1 mM EDTA, and 10 mM KHCO_3_). The remaining cells were cultured in complete medium (the serum from normal mice or tumor-bearing mice) supplemented with GM-CSF (10 ng/mL, 315–03, Peprotech) for 5 days. In a separate experiment, M-CSF (50 ng/mL, 315–02, Peprotech), CXCL1 (50 ng/mL, 250–11, Peprotech), or CXCL2 (50 ng/mL, 250–15, Peprotech) was added to the induction system. Next, 32D clone three cells transfected with PEGP-N1 (an empty vector), CXCR2, or CXCR2 and SAP18 were cultured in complete medium supplemented with GM-CSF (10 ng/mL) for 5 days. M-CSF (50 ng/mL), CXCL1 (50 ng/mL), or CXCL2 (50 ng/mL) was respectively added into the induction system.

### Construction of the lentiviral expression plasmid and gene transfection

Synthesized short hairpin (sh)RNAs or overexpression sequences were cloned into the vectors, and the constructed plasmids or shCtrl plasmid were transfected into HEK-293T cells, together with the packaging plasmid psPAX2 and the envelope plasmid, pMD2.G (both from Addgene) using Lipofectamine 2000 reagent (Invitrogen). To knock down SAP18 or overexpress SAP18 in tumor-bearing mice, the collected supernatant was concentrated and intrapleurally injected into three-week tumor-bearing mice four times every other day. To overexpress CXCR2 or SAP18 in 32D clone three cells, pEGFP-N1-CXCR2 or pEGFP-N1-SAP18 was electrotransfected into the cells. The relative sequences that were used are listed in Supplementary Table 1.

### Quantitative real-time PCR

Total RNA was extracted using TRIzol reagent (Invitrogen) and reverse transcribed into cDNA according to the manufacturer’s instructions (Thermo Scientific). Quantitative PCR was performed using SYBR Green Master Mix (Roche, Basel, Switzerland) and a Roche LightCycler 480 (Roche, Basel, Switzerland) real-time RT-PCR system. The level of mRNA expression was normalized to the housekeeping gene, β-actin, based on the ΔΔCt algorithm. The primer sequences that were used are listed in Supplementary Table 2.

### Flow cytometry analysis

Single-cell suspensions of bone marrow, spleen, and blood samples were prepared and stained as previously described ^13^. The bone marrow and spleens were ground and filtered through a 200-μm cell strainer. To eliminate the erythrocytes, single cell suspensions were treated with a hypotonic lysis buffer. The single cell suspensions were stained for 30 min at 4 °C with appropriate dilutions of various combinations of the following fluorochrome-conjugated antibodies: anti-CD11b-FITC (clone M1/70), anti-CD45-PE/Cy7 (clone 30-F11), anti-Ly6G-APC/Cy7 (clone 1A8), anti-Ly6C-PE (clone AL-21), anti-CXCR2-Alexa® Fluor 647 (clone SA044G4), anti-Lineage-Percp-Cy5.5 mouse lineage antibody cocktail, anti-CD117-BB515 (clone 2B8), anti-Sca1-PE/Cy7 (clone D7), anti-FcγRII/III-APC/Cy7 (clone 2.4G2), anti-CD135-PE (clone A2F10.1), anti-CD34-Alexa® Fluor 647(clone RAW34), anti-CD11b-biotin (clone M1/70), anti-CXCR2-PE (clone SA004G4), anti-CD115-PE/Cy7 (clone AFS98), and anti-CD115-APC (clone AFS98), which were all purchased from BD Biosciences, eBioscience or Biolegend. The cells were further fixed using 10% formaldehyde (Sigma Aldrich) for 10 min and permeabilized using 0.1% TritoX-100 (Sigma Aldrich) for 10 min, then stained for p-STAT3 (clone 13A31, PE/Cy5.5-conjugated, Biolegend) or Ki67 (clone 16A8, FITC-conjugated, Biolegend). For Annexin-V analysis, cells were stained according to the manufacturer’s instructions (BD Biosciences). The stained cells were acquired on a FACSCanto II (BD Biosciences) and the data were analyzed using FACSDiva software (BD Biosciences) and Flow Jo 7.6.1 software (Treestar). Dead cells and doublets were excluded based on the forward and side scatter.

### Western blot

The indicated cells were lysed in lysis buffer (150 mM NaCl, 50 mM Tris-HCl [pH 7.5], 1% NP-40, 1 mM EDTA, 0.5% sodium deoxycholate, 0.1% sodium dodecyl sulfate, 1 mM Na_3_VO_4_, 1 mM NaF, 1 mM PMSF, and 0.1 mg/mL leupeptin/aprotinin) on ice for 30 min, centrifugated at 15,000*×g* for 30 min, and the supernatants were collected for Western blotting. The primary antibodies were incubated with the membrane overnight at 4 °C. Horseradish peroxidase-conjugated goat anti-rabbit-mouse or rabbit IgG secondary antibodies (Beyotime, Haimen, China) were incubated with the nitrocellulose blotting membranes (GE Healthcare Life science, Boston, Massachusetts) for 1 h at room temperature in the next morning. Images were obtained using a chemiluminescence (ECL) detection system (ProteinSimple, San Jose, CA). Quantified band intensities were normalized using β-actin as housekeeping protein. Blots were scanned with a Tanon imaging system (5200, Shanghai, China). The primary antibodies used included anti-STAT3, anti-ERK1/2, anti-p-STAT3, anti-p-ERK1/2, anti- PI3Kγ, anti-HRAS, anti-SAP18, anti-JAK, anti-p-JAK, anti-SHP2, anti-p-SHP2, anti-PARP1, anti-PAR, and anti-β-actin, which were all purchased from Santa Cruz Biotechnology and Cell Signaling Technology.

### Isolation of mo- MDSCs from the blood and HSPCs from the bone marrow

To isolate mo-MDSCs, tumor-bearing mice were sacrificed by a tail vein injection with 4% EDTA. Blood was collected, and the erythrocytes were eliminated with a hypotonic lysis buffer. The remaining cells were collected and the mo-MDSCs were sorted with a Myeloid-Derived Suppressor Cell Isolation Kit using an AutoMACS sorter (Miltenyi Biotec, Germany) according to the manufacturer’s instructions. To isolate HSPCs, bone marrow cells were obtained from the femurs of mice and erythrocytes were eliminated using a hypotonic lysis buffer. First, the lineage^-^ cells were sorted using a Mouse Lineage Cell Depletion Kit (Miltenyi Biotec, Germany) using the AutoMACS sorter (Miltenyi Biotec, Germany) according to the manufacturer’s instructions. Then, lineage^-^ CD117^+^ cells were sorted from the lineage^-^ cells using a Mouse CD117 microbead kit (Miltenyi Biotec, Germany) using an AutoMACS sorter (Miltenyi Biotec, Germany) according to the manufacturer’s instructions. The lineage^-^CD117^+^ cells were HSPCs. The purity of the mo-MDSCs and HSPCs was assessed by flow cytometry and found to be ≥90%.

### Gene-expression analysis

For RNA sequencing (RNA-seq), 32D clone three cells transfected with CXCR2 or an empty vector were both incubated with CXCL1 and CXCL2 for 4 h and harvested. The cellular RNA was extracted using Trizol reagent followed by a genomic DNA elimination step. RNA purity was assessed using the kaiaoK5500® Spectrophotometer (Kaiao, Beijing, China). The RNA integrity and concentration was assessed using an RNA Nano 6000 Assay Kit of the Bioanalyzer 2100 system (Agilent Technologies, CA, USA). Library construction and sequencing on an Illumina HiSeq 2500 instrument was performed at Annoroad Gene Technology Corporation (Beijing, China). Bowtie2 v2.2.3 was used to build the genome index, and clean data was then aligned to the reference genome using HISAT2 v2.1.0. The level of gene expression was quantified using a software package called FPKM (Fragments Per Kilobase Millon Mapped Reads). DEGseq v1.18.0 was used for differential gene expression analysis between two samples with non-biological replicates. Genes that were consistently 1.5-fold upregulated or two-fold downregulated in RNA sequencing are listed in Supplementary Table 3. RNA sequencing of the raw data and processed expression data for this study have been deposited in the NCBI Gene Expression Omnibus and are accessible through GEO Series accession number: [GEO: GSE125347].

### Quantitative chromatin immunoprecipitation (ChIP) assay

HSPCs were chemically cross-linked with a 1% formaldehyde solution for 10 min at room temperature with gentle agitation and quenched with 0.125 M glycine. The fixed cells were resuspended, lysed, and sonicated to solubilize and shear the crosslinked DNA. The samples were ultra-sonicated for 8 min with 30 s ultra-sonication at 30 s intervals. The resulting fragmented chromatin extract was precleared with protein A/G beads (ThermoFisher, Beijing, China) and then incubated separately overnight with SAP18 (Santa Cruz Biotechnology) or mSin3A (Santa Cruz Biotechnology) antibodies followed by washing, elution, and reverse cross-linking. DNA was purified using PCR purification kits (QIAGEN, Hilden, Germany) and amplified by quantitative PCR using the specific primer sets as listed below for individual genes. ChIP-qPCR calculations were performed as described previously^52^. In brief, the protein-specific Ab ChIP-ed DNA signal intensity value was divided by the intensity value of the IgG-ChIP-ed signal, representing the fold enrichment of the protein on the specific region of genomic DNA. The primer sequences used are listed in Supplementary Table 4.

### Statistical analysis

Statistical analysis was performed using Prism 5 software (GraphPad software, La Jolla, CA, USA). Data correspond to the mean ± SD. Results were analyzed using paired *t*-test, or a one- or two-way ANOVA (analysis of variance) followed by post hoc tests (see legends). *P* values were considered statistically significant when *p* < 0.05 (*p < 0.05; **p < 0.01; and ***p < 0.001; ns, not significant).

## Supplementary information

Description of Supplementary Figure and Supplementary Figure

Supplementary Table 1 Primer sequences of shRNAs and overexpression

Supplementary Table 2 Primer sequences of qPCR

Supplementary Table 3 Differentially expressed genes of RNA-seq

Supplementary Table 4 Primer sequences of ChIP
