# Correction: CXCR2 expression on granulocyte and macrophage progenitors under tumor conditions contributes to mo-MDSC generation via SAP18/ERK/STAT3

**DOI:** 10.1038/s41419-019-1869-6

**Published:** 2019-09-05

**Authors:** Xiaoqing Han, Huifang Shi, Yingying Sun, Chao Shang, Tao Luan, Dake Wang, Xueqing Ba, Xianlu Zeng

**Affiliations:** 1 The Key Laboratory of Molecular Epigenetics of Ministry of Education, Institute of Genetics and Cytology, School of Life Science, Northeast Normal University, Changchun, Jilin China; 2 People’s Hospital of Rizhao, Rizhao, China

**Keywords:** Cancer microenvironment, Immune evasion

**Correction to: Cell Death and Disease**

10.1038/s41419-019-1837-1, Published online 08 July 2019.

Following publication of this article, it was realized that the ordering of the last two authors was reversed and required correction. The correct ordering of the authors for the paper is as follows:

Xiaoqing Han, Huifang Shi, Yingying Sun, Chao Shang, Tao Luan, Dake Wang, Xueqing Ba (*) & Xianlu Zeng (*)

[(*) indicates corresponding author].

This has been corrected in both the PDF and HTML versions of the Article.

We apologize for any inconvenience this may have caused the readers.

