# Supplementary material for: CXCR2 expression on granulocyte and macrophage progenitors under tumor conditions contributes to mo-MDSC generation via SAP18/ERK/STAT3
Source: Cell Death Dis. 2019 Aug 8;10(8):598. doi: 10.1038/s41419-019-1837-1 (PMC6687752; doi:10.1038/s41419-019-1837-1)

**Supplementary figure legends**

**Figure. S1** **The normal myelopoiesis was analyzed in WT and CXCR2-/- normal mice. (A-C)** The number of myeloid cells (CD11b^+^Ly6C^+^ cells, CD11b^+^Ly6G^+^ cells, CD11b^+^CD11c^+^ cells) and other immunomodulatory cells (CD4^+^T cells, CD8^+^T cells, CD119^+^B cells and natural killer [NK] cells) in the blood (Fig. S1A), bone marrow (Fig. S1B) and spleen (Fig. S1C) of wild type normal mice and CXCR2-/- normal mice were also detected by flow cytometry. **(D)** The number of total bone marrow cells, LKs (Lin^-^Sca-1^-^c-Kit^+^), CMPs (LK, FcγRII/III^int^CD34^+^), GMPs (LK, FcγRII/III^hi^ CD34^+^), and MDPs (Lin^-^CD117^+^CD115^+^CD135^+^Ly6C^−^CD11b^−^) and the proliferative activity of CMPs, GMPs and MDPs were examined in wild type normal mice and CXCR2-/- normal mice by flow cytometry. Bars represent the mean ± SD of ﬁve independent experiments. One-way ANOVA with repeated measures followed by Dunnett’s post hoc test or two-way ANOVA followed by Holm–Sidak’s post hoc test show the statistical signiﬁcance (*p < 0.05, **p < 0.01, and ***p < 0.001; ns, not significant).

**Figure. S2 The proliferation, apoptosis and differentiation of mo-MDSCs were analyzed in WT and CXCR2−/− mice. (A and B)** The expression of Ki67 of mo-MDSCs in the bone marrow and spleens of WT or CXCR2-/- mice was analyzed by flow cytometry. **(C and D)** Annexin V expression of mo-MDSCs in the spleen and blood of WT or CXCR2−/− mice was analyzed by flow cytometry. **(E)** The differentiation of mo-MDSCs into CD11b^+^CD11c^+^ and CD11b^+^ F4/80^+^ cells in vitro was analyzed by flow cytometry. The mo-MDSCs were isolated from the blood of WT or CXCR2-/- tumor-bearing mice and treated with GM-CSF for three or five days. **(F)** Quantitative analyses of mo-MDSCs differentiation shown in (E). The bars represent the mean ± SD of ﬁve independent experiments. A one-way ANOVA with repeated measures followed by a Dunnett’s post-hoc test or two-way ANOVA followed by a Holm-Sidak’s post-hoc test show the level of statistical signiﬁcance (*p < 0.05; **p < 0.01; and ***p < 0.001; ns, not significant).

**Figure. S3 (A)** The expression of Lineage, CD117, Sca1 and CXCR2 on 32D clone 3 cells was analyzed by flow cytometry. **(B)** The transfection efficiency of CXCR2 to 32D clone 3 cells was analyzed by flow cytometry. The 32D clone 3 cells were transfected with CXCR2, and normal 32D clone 3 cells were used as a control.

**Figure. S4** The expression of PARP1, PAR, JAK2, p- JAK2, SHP2, and p-SHP2 was analyzed by Western blot in the HSPCs of control or tumor-bearing (TB) mice. The HSPCs were sorted from either control or tumor-bearing mice.

**Figure. S5 The ERK1/2 phosphorylation and the percentage of mo-MDSCs were analyzed in mice treated with isoproterenol. (A)** The expression of ERK1/2 and p-ERK1/2 in HSPCs was analyzed by Western blot. CXCR2-/- normal mice were randomly treated with isoproterenol (15 mg.g^−1^, day^−1^, suspended in 50 μL soy bean oil, Sigma Aldrich, USA) or a vehicle daily for 1, 3, 5, 7, or 9 days. At the end of the treatment period, the animals were sacrificed and HSPCs were sorted from the treated mice. **(B)** Mice were treated as described in (A). The percentage of mo-MDSCs in the bone marrow was evaluated by flow cytometry. **(C)** CXCR2-/- tumor-bearing mice were randomly treated with isoproterenol daily for seven days. The level of ERK1/2 and p-ERK1/2 expression was analyzed by Western blot in HSPCs. **(D)** Mice were treated as described in (C). The percentage of mo-MDSCs in the bone marrow was evaluated by flow cytometry. Bars represent the mean ± SD of ﬁve independent experiments. One-way ANOVA with repeated measures followed by Dunnett’s post hoc test or two-way ANOVA followed by Holm–Sidak’s post hoc test show the statistical signiﬁcance (*p < 0.05, **p < 0.01, and ***p < 0.001; ns, not significant).

**Figure. S6** The level of *Pu.1* and *Egr1* mRNA was evaluated in 32D clone 3 cells by qPCR. The cells were transfected with CXCR2 and SAP18 and subsequently incubated with M-CSF, CXCL1, or CXCL2 for five days in the presence of GM-CSF. 32D clone 3 cells transfected with CXCR2 were used as a control. Bars represent the mean ± SD of ﬁve independent experiments.

**Figure. S7** **(A-D)** Quantitative analyses of the protein in Fig. 6A (Fig. S7A), 6C (Fig. S7B), 6E (Fig. S7C), 6J (Fig. S7D) by Image J software. SAP18, HRAS and PI3Kγ were normalized over β-actin, p-ERK1/2 was normalized over ERK1/2, and p-STAT3 was normalized over STAT3. Bars represent the mean ± SD of ﬁve independent experiments. One-way ANOVA with repeated measures followed by Dunnett’s post hoc test or two-way ANOVA followed by Holm–Sidak’s post hoc test show the statistical signiﬁcance (*p < 0.05, **p < 0.01, and ***p < 0.001; ns, not significant).

**Figure. S8** **The percentage of mo-MDSCs and G-MDSCs were evaluated in the blood. (A)** Flow cytometry analysis of the percentage of mo-MDSCs and G-MDSCs in the blood from wild-type control, CXCR2-/- control, wild-type tumor-bearing, SAP18 overexpressing WT tumor-bearing, CXCR2-/- tumor-bearing, and shSAP18 CXCR2-/- tumor-bearing mice. **(B and C)** Quantitative analyses of the data presented in (A). Bars represent the mean ± SD of ﬁve independent experiments. One-way ANOVA with repeated measures followed by Dunnett’s post hoc test or two-way ANOVA followed by Holm–Sidak’s post hoc test show the statistical signiﬁcance (*p < 0.05, **p < 0.01, and ***p < 0.001; ns, not significant).

Figure. S9 The expression of STAT3 and p-STAT3 were analyzed by Western blot in 32D clone 3 cells transfected with CXCR2 and incubated with Stattic (1μM, 3μM, 5μM) for 2h. The treated cells were incubated with M-CSF (50 ng/mL).

**Supplementary figure**

Figure S1

A


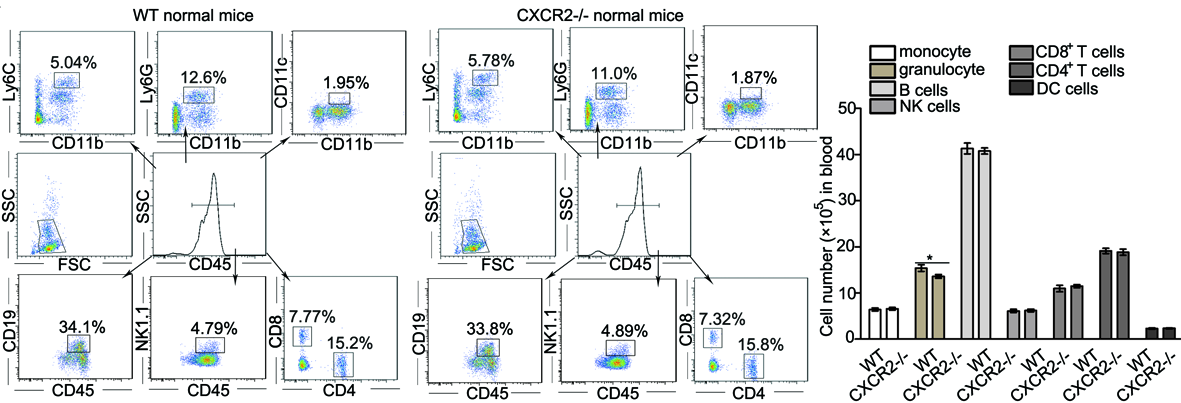


B


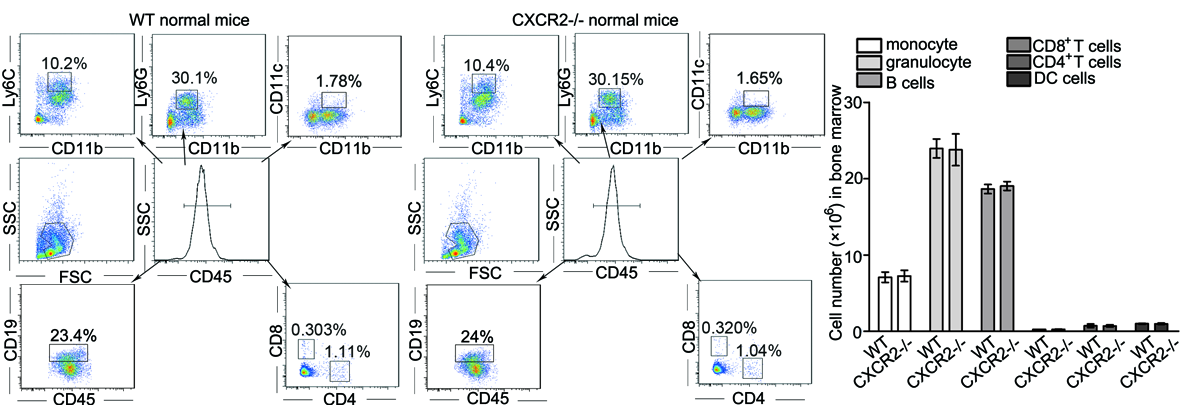


C


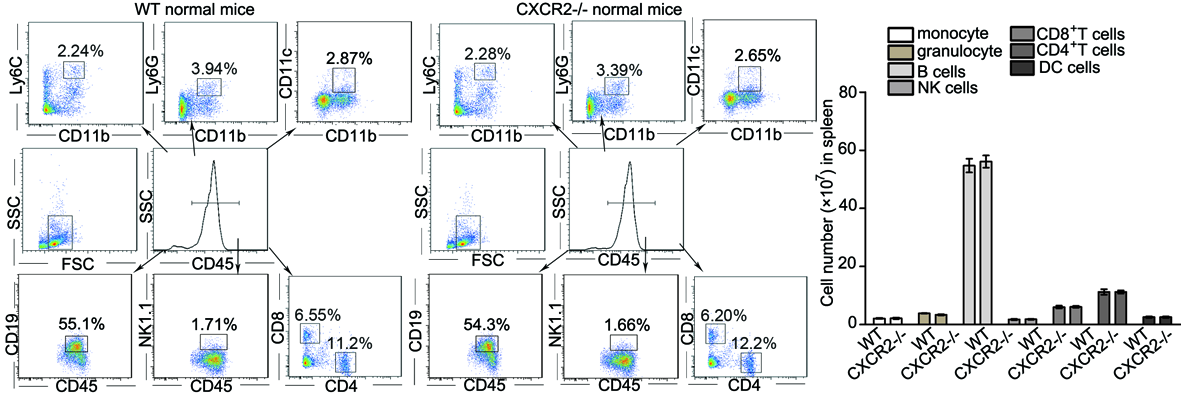


D


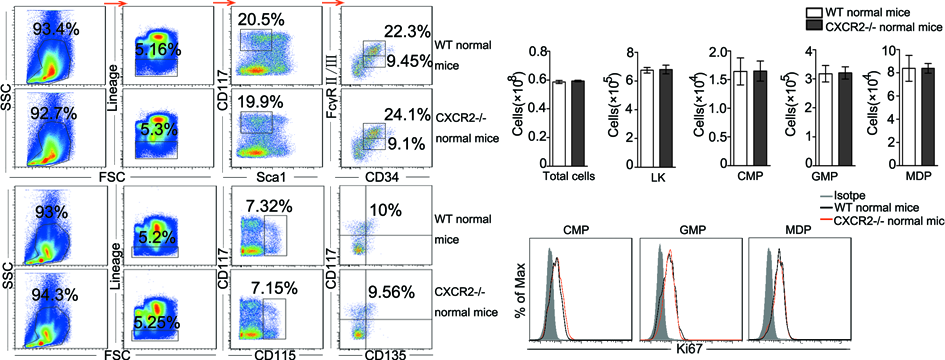


Figure S2


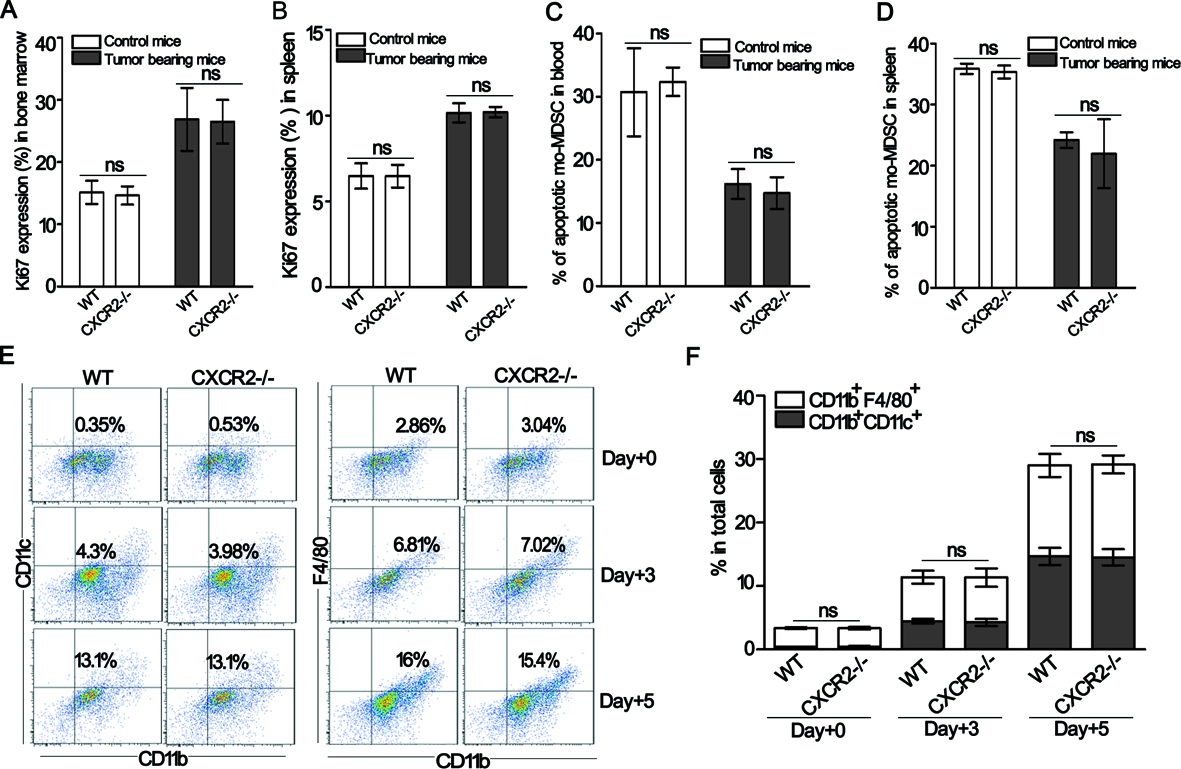


Figure S3


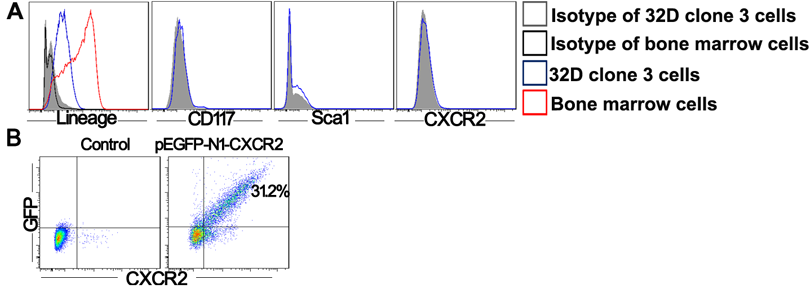


Figure S4


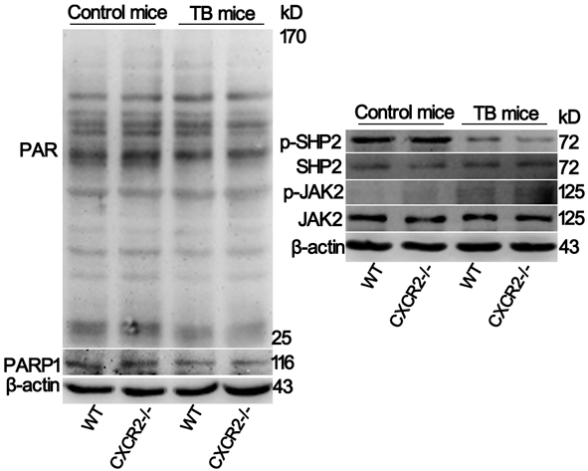


Figure S5


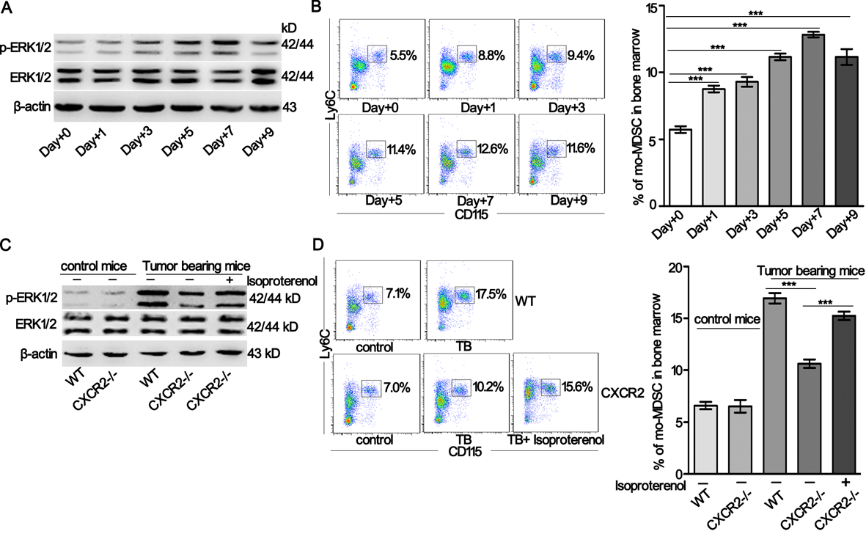


Figure S6


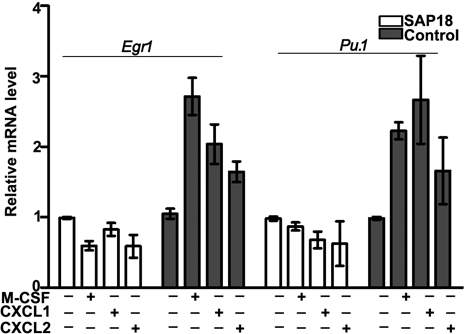


Figure S7


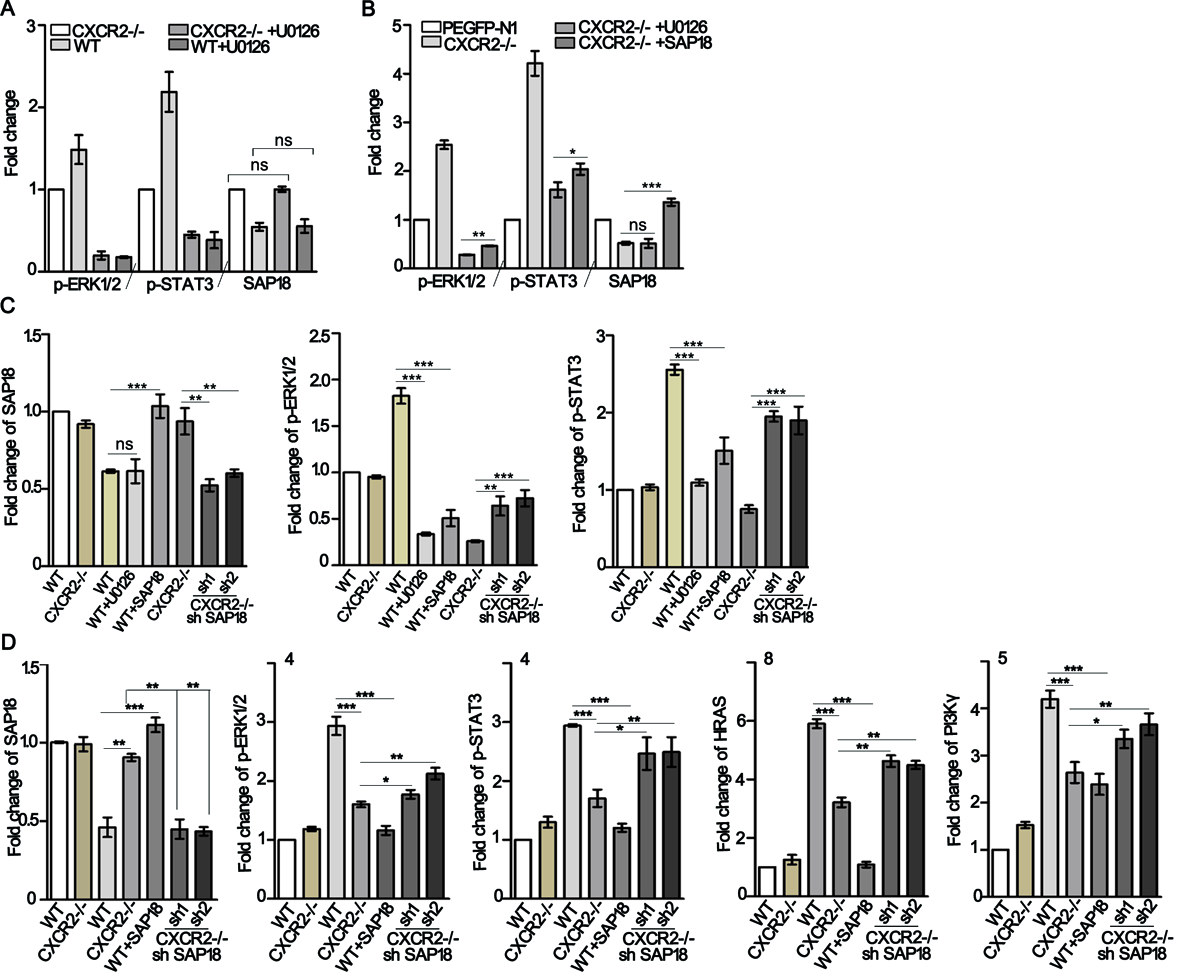


Figure S8


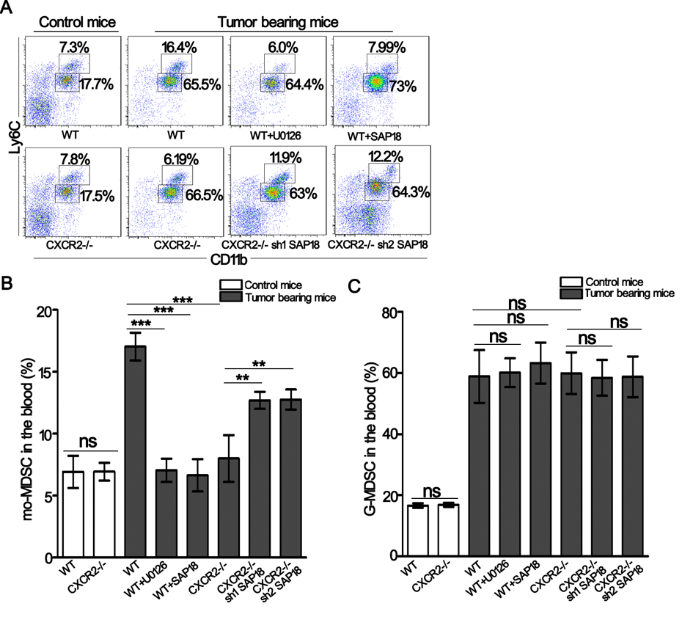


Figure S9


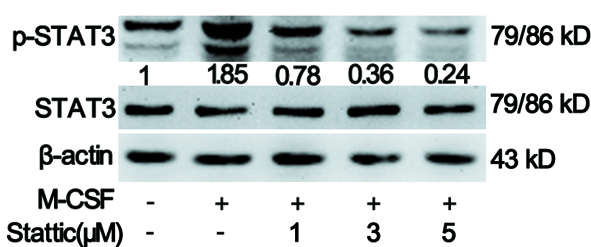

Supplement: Supplementary file 1 — Description of Supplementary Figure and Supplementary Figure [file 41419_2019_1837_MOESM1_ESM.docx]
