# Supplementary material for: CXCR2 expression on granulocyte and macrophage progenitors under tumor conditions contributes to mo-MDSC generation via SAP18/ERK/STAT3
Source: Cell Death Dis. 2019 Aug 8;10(8):598. doi: 10.1038/s41419-019-1837-1 (PMC6687752; doi:10.1038/s41419-019-1837-1)
Supplement: Supplementary file 2 — Supplementary Table 1 Primer sequences of shRNAs and overexpression [file 41419_2019_1837_MOESM2_ESM.docx]

**Supplementary Table 1**

Primer sequences of shRNAs and overexpression

| Primer name | Forward Primer(5’-3’) | Reverse Primer(5’-3’) |
| --- | --- | --- |
| sh1-SAP18 | CGCGTCCCCGCGTTACCC  AGGAGGAAATTTCAAGA  GAATTTCCTCCTGGGT  AACGCTTTTTGGAAAT | CGATTTCCAAAAAGCG  TTACCCAGGAGGAAATT  CTCTTGAAATTTCCTCC  TGGGTAACGCGGGGA |
| sh2-SAP18 | CGCGTCCCCGGATGGAT  GCAACCTTGAATTCAA  GAGATTCAAGGTTGCA  TCCATCCTTTTTGGAAAT | CGATTTCCAAAAAGGATGG  ATGCAACCTTGAATCTC  TTGAATTCAAGGTTGC  ATCCATCCGGGGA |
| CXCR2 | CCCAAGCTTGCCACCATGG  GAGAATTCAAGGTGGAT | GGGGTACCGTGAGGGTA  GTAGAGGTGTTTGC |
| SAP18 | CCCAAGCTTGCCACCAT  GGCGGTGGAGTCGCGCGTT | GGGGTACCGTGTAGGGT  CTCATCCTCCCTGA |

Primer sequences of shRNAs were obtained using BLOCK-iT™ RNAi Designer of Invitrogen and the overexpression sequences were obtained using primer premier 5.
