# Supplementary material for: CXCR2 expression on granulocyte and macrophage progenitors under tumor conditions contributes to mo-MDSC generation via SAP18/ERK/STAT3
Source: Cell Death Dis. 2019 Aug 8;10(8):598. doi: 10.1038/s41419-019-1837-1 (PMC6687752; doi:10.1038/s41419-019-1837-1)
Supplement: Supplementary file 3 — Supplementary Table 2 Primer sequences of qPCR [file 41419_2019_1837_MOESM3_ESM.docx]

**Supplementary Table 2**

Primer sequences of qPCR

| Gene | Forward Primer(5’-3’) | Reverse Primer(5’-3’) |
| --- | --- | --- |
| *β-actin* | AACAGTCCGCCTAGAAGCAC | CGTTGACATCCGTAAAGACC |
| *CXCR2* | CCTCAAACGGGATGTATT | GCTCTGTCACCGATGTCT |
| *Arg1* | GACAGGGCTCCTTTCAGGAC | CTTGGGAGGAGAAGGCGTTT |
| *iNOS* | GCATGGAACAGTAT  AAGGCAAACA | GTTTCTGGTCGATG  TCATGAGCAA |
| *Hoxa5* | CCTACACTCGCTACCAGACCCT | GCGGCCATACTCATGCTTTT |
| *Hoxa7* | CTTCCTCCTCTTCTTCCTCCTCTT | GCCAGTTTCCGCATCTACCC |
| *Evil1* | GGTAGAGCCAGTGGGACAA | TTGCGGGTGAAACAAGAAT |
| *Meis1* | GGTAAGTCCTGTATCTTGTGCC | TCTAACTGACCAGCCCTCTTG |
| *Pu.1* | TTGGACGAGAACTGGAAGGT | CAGATGAGGAGGAGGGTGA |
| *Egr1* | GGATGAAGAGGTCGGAGGA | CAGCGGCGGTAATAGCAG |

Primer sequences were obtained using primer premier 5.
