# Supplementary material for: CXCR2 expression on granulocyte and macrophage progenitors under tumor conditions contributes to mo-MDSC generation via SAP18/ERK/STAT3
Source: Cell Death Dis. 2019 Aug 8;10(8):598. doi: 10.1038/s41419-019-1837-1 (PMC6687752; doi:10.1038/s41419-019-1837-1)
Supplement: Supplementary file 4 — Supplementary Table 3 Differentially expressed genes of RNA-seq [file 41419_2019_1837_MOESM4_ESM.docx]

**Supplementary Table 3**

Differentially expressed genes of RNA-seq

| T1-C1 | | T3-C3 | |
| --- | --- | --- | --- |
| up | down | up | down |
| Gm43844 | SAP18 | Gm23935 | Gm10800 |
| Ripk3 | Gm28438 | Lars2 | Gm5620 |
| Nynrin | Gm38082 | CT010467.1 | SAP18 |
| Gm42748 | A430110C17Rik | Gm24270 | Gm26870 |
| Gm14400 | Hspd1-ps3 | Gm24187 | Gm17383 |
| Gm11346 | Gm45779 | Krt14 | P2ry6 |
| Gm12715 | Gm29216 | Krt1 | C920006O11Rik |
| Cacna1a | Gm45553 | Olfr1152 | Gm15232 |
| Gm44836 | Prr7 | Krt16 | A230060F14Rik |
| Zfp786 | B130021K23Rik | Rptn | Eng |
|  | Alyref2 | Krt6b | Gm29216 |
|  | Aspn | Krt6a | Rab3a |
|  | Gm8850 | Fgf23 | AC140354.1 |
|  |  | Krt5 |  |
|  |  | Asprv1 |  |
|  |  | AC121793.2 |  |
|  |  | Gm43961 |  |
|  |  | Sarm1 |  |
|  |  | Flg |  |
|  |  | A430105J06Rik |  |
|  |  | Mroh8 |  |
|  |  | Gm11847 |  |
|  |  | Perp |  |
|  |  | Dmkn |  |
|  |  | AC140448.1 |  |
|  |  | Gm12355 |  |
|  |  | Fbxl13 |  |
|  |  | 4833417C18Rik |  |
|  |  | Acvrl1 |  |
|  |  | Calm4 |  |
|  |  | Gm22767 |  |
|  |  | 1600002K03Rik |  |
|  |  | Gm26964 |  |
|  |  | Chil1 |  |
|  |  | 5033403F01Rik |  |
|  |  | Papolb |  |
|  |  | Gm44685 |  |
|  |  | Emc9 |  |

T1 and T3 represent the 32D clone 3 cells transfected with CXCR2, C1 and C3 represent the 32D clone 3 cells transfected with an empty vector.
