# Supplementary material for: CXCR2 expression on granulocyte and macrophage progenitors under tumor conditions contributes to mo-MDSC generation via SAP18/ERK/STAT3
Source: Cell Death Dis. 2019 Aug 8;10(8):598. doi: 10.1038/s41419-019-1837-1 (PMC6687752; doi:10.1038/s41419-019-1837-1)
Supplement: Supplementary file 5 — Supplementary Table 4 Primer sequences of ChIP [file 41419_2019_1837_MOESM5_ESM.docx]

**Supplementary Table 4**

Primer sequences of ChIP

| Gene | Forward Primer(5’-3’) | Reverse Primer(5’-3’) |
| --- | --- | --- |
| KRAS | AGTCTACAAGAGCGGGCAGA | TCTTGGCAGCCATTCTTTCT |
| HRAS | CAATTGGCTCATCGAAAGGT | GCCAAGAGCATGGAGAGAAC |
| NRAS | ACAACTGCTTGCCTTCTTCC | CTTGGCTGACGAAGAACCAT |
| PI3Kγ | TGCTTCTCCAGCAGTCATTG | TTTTCCAGCAGCCTCCTTT |

Primer sequences were obtained using primer premier 5.
